# GREM1/PPP2R3A expression in heterogeneous fibroblasts initiates pulmonary fibrosis

**DOI:** 10.1186/s13578-022-00860-0

**Published:** 2022-08-06

**Authors:** Xiaoni Shi, Jing Wang, Xinxin Zhang, Shaoqi Yang, Wei Luo, Sha Wang, Jie Huang, Mengling Chen, Yusi Cheng, Jie Chao

**Affiliations:** 1 Department of Physiology, School of Medicine, Southeast University, 87 Dingjiaqiao Rd, Nanjing, 210009 Jiangsu China; 2 Key Laboratory of Environmental Medicine Engineering, Ministry of Education, School of Public Health, Southeast University, Nanjing, 210009 Jiangsu China; 3 Jiangsu Provincial Key Laboratory of Critical Care Medicine, School of Medicine, Zhongda Hospital, Southeast University, Nanjing, 210009 Jiangsu China; 4 School of Medicine, Xizang Minzu University, Xianyang, 712082 Shanxi China

**Keywords:** Pulmonary fibrosis, Heterogeneous fibroblasts, Single-cell transcriptomics, Spatial transcriptomics

## Abstract

**Background:**

Fibroblasts have important roles in the synthesis and remodeling of extracellular matrix (ECM) proteins during pulmonary fibrosis. However, the spatiotemporal distribution of heterogeneous fibroblasts during disease progression remains unknown.

**Results:**

In the current study, silica was used to generate a mouse model of pathological changes in the lung, and single-cell sequencing, spatial transcriptome sequencing and an analysis of markers of cell subtypes were performed to identify fibroblast subtypes. A group of heterogeneous fibroblasts that play an important role at the early pathological stage were identified, characterized based on the expression of inflammatory and proliferation genes (termed inflammatory-proliferative fibroblasts) and found to be concentrated in the lesion area. The expression of GREM1/protein phosphatase 2 regulatory subunit B''alpha (PPP2R3A) in inflammatory-proliferative fibroblasts was found to initiate early pulmonary pathological changes by increasing the viability, proliferation and migration of cells.

**Conclusions:**

Inflammatory-proliferative fibroblasts play a key role in the early pathological changes that occur in silicosis, and during this process, GREM1 is the driving factor that targets PPP2R3A and initiates the inflammatory response, which is followed by irreversible fibrosis induced by SiO_2_. The GREM1/PPP2R3A pathway may be a potential target in the early treatment of silicosis.

**Supplementary Information:**

The online version contains supplementary material available at 10.1186/s13578-022-00860-0.

## Introduction

The inhalation of free crystalline silica or silica occurs in many industries [1] and may lead to fibroblast activation and excessive accumulation and deposition of extracellular matrix (ECM) [2]. Lung function impairment increases as the disease progresses and becomes progressively worse over time even if the patients are no longer being exposed. The diagnosis of pulmonary fibrosis caused by silica inhalation is usually based on the exposure to a high level of silica dust and radiological characteristics; similar diseases [3], such as powdery tuberculosis, idiopathic pulmonary fibrosis (IPF), other lung interstitial diseases and cancers, must be excluded. Effective treatments for the disease are lacking, and the exact pathogenesis remains unclear.

Pulmonary fibroblasts (PFBs) are the main pulmonary interstitial cells [4]. These important effector cells are involved in the damage and repair processes in the body and are maintained in a resting state under physiological conditions (resting) but respond rapidly under pathological conditions. The proliferation of these cells completes the repair of cellular damage and restores the characteristics of the resting state. These cells participate in fibrosis through proliferation, migration and the synthesis and release of matrix materials such as collagen. Cell injury, infection or other stimuli promote the differentiation of mesenchymal cells into activated or pathological fibroblasts, and these cells induce inflammation [5]. The heterogeneity of lung fibroblasts indicates that these cells may be derived from different cell types, may represent different activation stages, or may be affected by the microenvironment [6]. The different responses of heterogeneous fibroblasts to the internal environment may be the main cause of pulmonary fibrosis as well as the main cause of lung cancer and other cancers [7], and related studies have shown that the physiological and pathological types of fibroblasts display differences in gene expression and cell surface markers [8–12].

In this study, we generated all major cell types based on single-cell RNA sequencing (scRNA-Seq) and developed a molecular map of fibroblasts to comprehensively elucidate the changes in the fibroblast types and spatial locations during pulmonary fibrosis. Through spatial transcriptomics combined with new calculation methods, we identified the localization of the different cell types. A quasi-chronological analysis was performed to determine the source and localization of the cells. Based on these results, the newly emerging heterogeneous fibroblasts express gremlin 1, DAN family BMP antagonist (GREM1), a member of the TGF-β superfamily that promotes cell proliferation and migration and is associated with pulmonary, liver, eye, and skin fibrosis. Protein phosphatase 2A regulatory subunit B ′′α (PPP2R3A) regulates the cell cycle mainly by targeting cell cycle regulators and apoptosis factors and has attracted attention due to its involvement in the regulation and development of tumor signaling pathways. PPP2R3A can modulate GREM1 to participate in early pathological changes in the lung that can potentially lead to the activation, proliferation and migration of fibroblasts.

## Results

### scRNA-seq classification of mouse lung fibroblasts

**Fig. 1:**
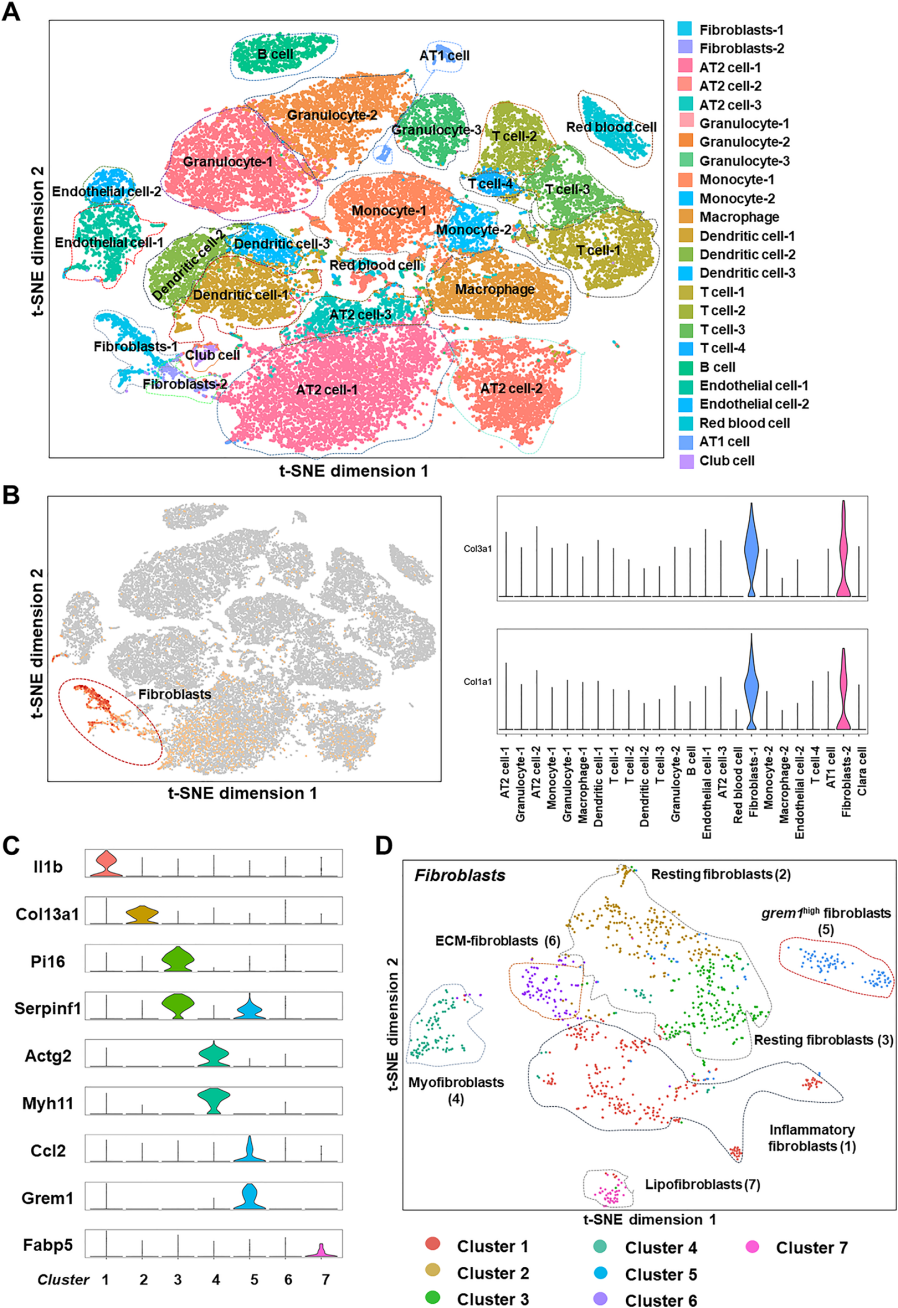
Classification of fibroblasts using single-cell transcriptome sequencing. **A** Single-cell sequencing divided all the cells in lung tissue into 24 subtypes. **B** Fibroblasts were separated based on Col1a1 and Col3a1 expression. **C** A marker gene used for the subtyping of fibroblasts. **D** Fibroblasts were subdivided into seven subtypes

According to recent reports, heterogeneous lung fibroblasts may be the main cause of lung fibrosis and early pathological changes [6, 13]. Before comprehensive identification and definition of the fibroblast subpopulations in normal and fibrotic lung tissues, we first grouped all the cells in lung tissue. C57BL/6 mice (20–25 g) were treated with silica, and after successful establishment of the model, the lung tissue was removed, and cells extracted from the lung tissue homogenate were subjected to single-cell sequencing using the 10 × Genomics Chromium platform. Through t-distribution stochastic neighborhood embedding (t-SNE) projection, the cells were visualized in two dimensions according to the expression profile, and all cells in the lung tissue were divided into 24 types according to the surface markers expressed on different cells (Fig. 1A). Among these cells, fibroblasts were separated by their specific markers Col3a1 and Col1a1 [14, 15] (Fig. 1B), and we then subdivided the fibroblasts into subtypes using different known fibroblast markers [6, 13, 16–18]. Cluster 6 was defined by a GO enrichment analysis of the molecular functions and biological processes of the top 50 genes in this subtype (Fig. 1C, D and S1A-C). The cells were defined as different subtypes, but Cluster 5 was unable to be defined accurately using existing gene markers. Due to the characteristic expression of the proliferation-related gene *grem1* in this subtype (this subtype exhibited the highest expression level of *grem1* among the subtypes), the cells in Cluster 5 were temporarily defined as *grem1*^high^ fibroblasts.

### Analysis of fibroblast subtypes

**Fig. 2:**
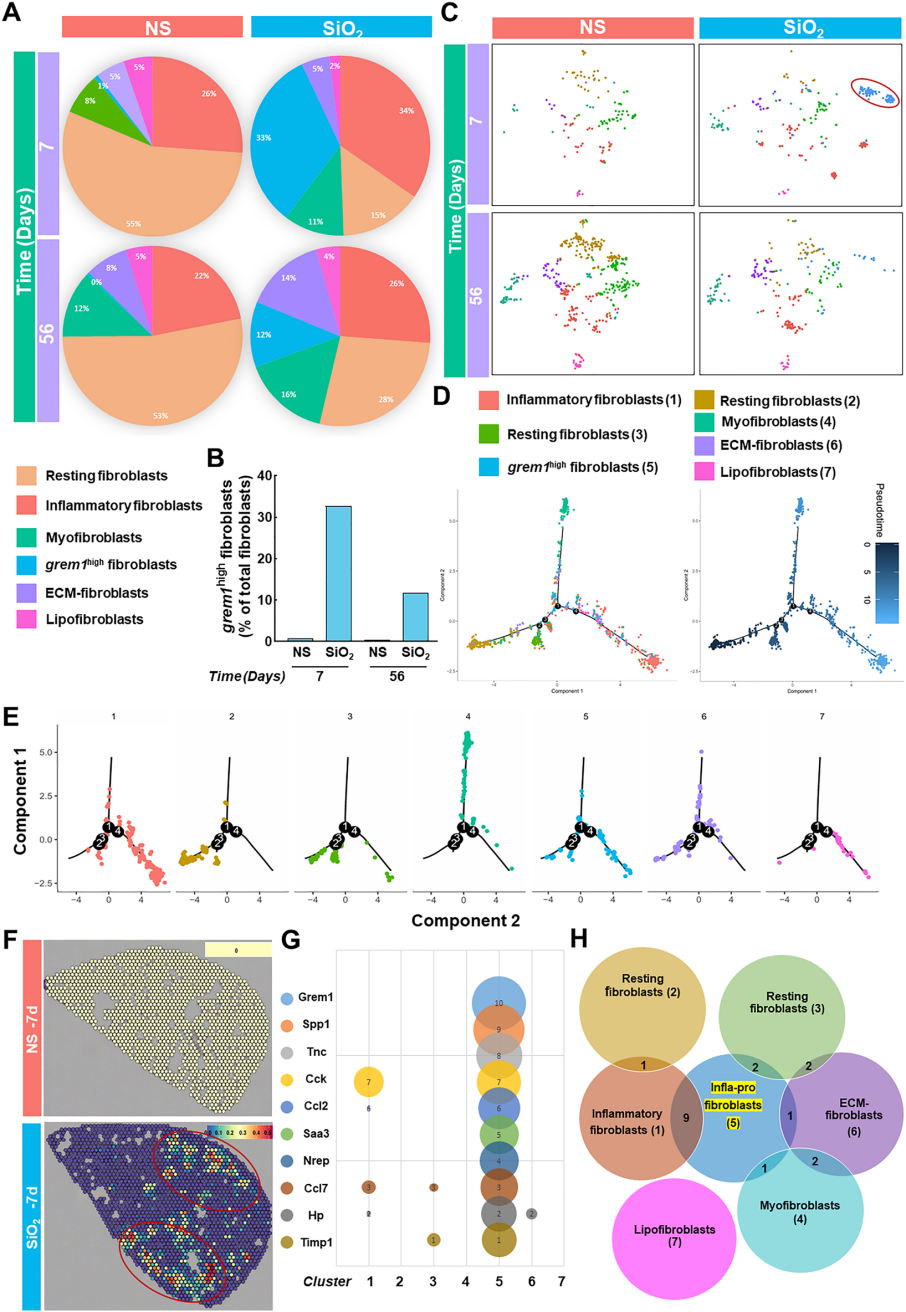
Correlation analysis of fibroblast subtypes. **A** Percentage of each fibroblast subtype among the total fibroblasts in different groups (saline and silica groups) at different time points (7 and 56 days). **B** Proportion of *grem1*^*high*^ fibroblasts in different groups at different time points. **C** Distribution of each subtype of fibroblasts in the saline and silica groups at 7 and 56 days. **D** The quasi-chronological analysis shows the status of the whole fibroblast population at each node of transdifferentiation. **E** Status of each subtype of fibroblasts at the node of transdifferentiation. **F** The lesion area contains a large number of *grem1*^*high*^ fibroblasts. **G** Based on the expression of the top 10 genes with multiple changes in Cluster 5 compared with other subtypes, the numbers 1–10 correspond to the 10 genes in the left column, and the bubble size represents the size of multiple changes. *grem1* is only expressed in Cluster 5. **H** Venn diagram showing the number of identical genes expressed in the different subtypes

Based on the aforementioned subtype definitions and the single-cell sequencing results of the lung tissues from mice in the saline and silica groups at 7 and 56 days obtained in the laboratory, the cell percentages of different groups and subgroups were analyzed (Fig. 2A). The percentage of subtype fibroblasts in each group is shown in Table 1. The *grem1*^high^ fibroblasts appeared specifically in the silica group, and the proportion detected at 7 days was higher than that detected at 56 days (Fig. 2B and C), which suggested that this subtype of cells plays a vital role at the early pathological stage. We then analyzed the source and destination of *grem1*^high^ fibroblasts through a pseudo-chronological sequence and found that these special heterogeneous fibroblasts originate from resting fibroblasts; after performing their functions, these cells transdifferentiate into myofibroblasts and inflammatory fibroblasts (Fig. 2D and E). We found that the *grem1*^high^ fibroblasts were primarily expressed in the focus area in the spatial map (Fig. 2F). We constructed a bubble chart to further determine whether the top 10 genes expressed by cells in Cluster 5 were also expressed at high levels in other subtypes and found that most genes were specifically expressed in the Cluster 5 subtype, but *grem1* was expressed at high levels only in this subtype (Fig. 2G). By constructing a Venn diagram, we then found that the top 50 genes expressed in *grem1*^high^ fibroblasts overlapped with those expressed in inflammatory fibroblasts, ECM fibroblasts, and myofibroblasts (Fig. 2H). Thus, this subtype of fibroblasts was termed inflammatory-proliferative fibroblasts.

**Table 1:** Statistical analysis of cell numbers for different subpopulations of fibroblasts

	Inflammation	Resting^1^	Myo	Pro-inflammatory	ECM	Lipo
NS-7 days	26.12	55.22	7.46	0.75	5.22	5.22
SiO_2_-7 days	34.63	14.79	10.89	32.68	5.06	1.95
NS-56 days	21.92	52.97	12.10	0.46	7.76	4.79
SiO_2_-56 days	26.20	27.51	15.72	11.79	14.41	4.37

Shows the numbers of 6 subgroups of fibroblasts in different groups at different time points (two groups of resting fibroblasts were combined into one group)

### Inflammatory-proliferative fibroblast-related bioinformatics analysis and scRNA-Seq verification

**Fig. 3:**
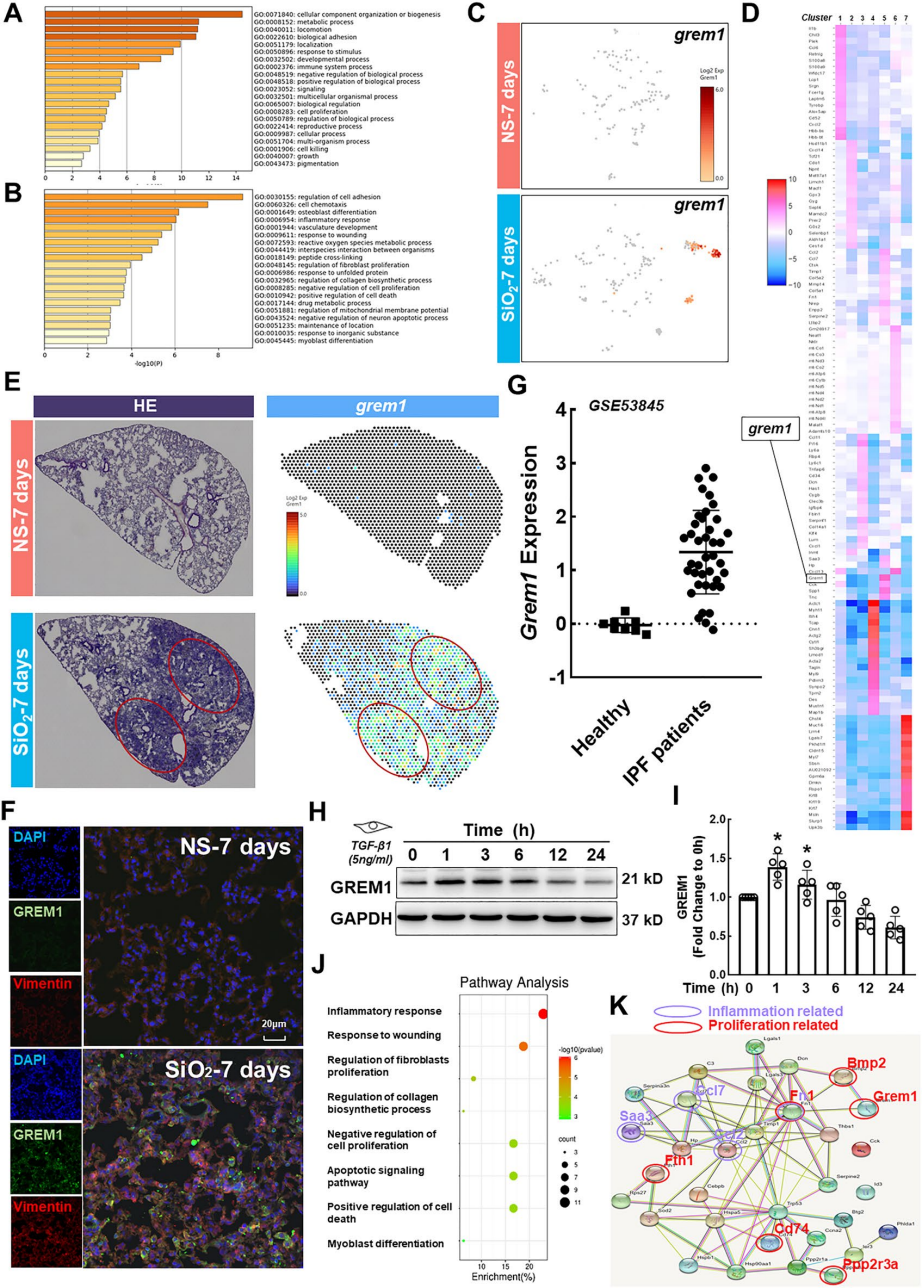
Results of biochemical analyses and validation of the scRNA-Seq analysis of inflammatory-proliferative fibroblasts. **A** GO enrichment analysis of the molecular functions of the top 50 genes in inflammatory-proliferative fibroblasts. **B** GO enrichment analysis of signaling pathways related to the top 50 genes in inflammatory-proliferative fibroblasts. **C** Expression of *grem1* in seven subtypes. **D** Gene heatmap of the seven subtypes. **E** The spatial transcriptomic sequencing results show that *grem1* is expressed at high levels in the lesion area. **F** Immunohistochemical staining showing that GREM1 is expressed on fibroblasts. The expression level of the experimental group was higher than that of the control group; the scale bar represents 20 μm. **G** In the GEO database, the expression of *Grem1* in the lung tissue of patients with IPF was significantly higher than that found in the healthy group (*p < 0.05). **H**, **I** In HPF-a cells, GREM1 expression first increased and then decreased over time, and the expression levels at 1 and 3 h were significantly different from those at 0 h (*p < 0.05). **J** The bubble chart shows the pathways of interest among the related pathways identified in inflammatory-proliferative fibroblasts. **K** Interaction map between proteins enriched in signaling pathways examined in this study

Based on the above-described analysis, we focused on inflammatory-proliferative fibroblasts and the characteristic expression of *grem1*. By enriching the top 50 genes in this group and analyzing their functions (Fig. 3A), we found that these genes play an important role in regulating biological processes, cell growth and proliferation. A GO enrichment analysis showed that the genes were enriched in cell adhesion, inflammation, cell death, myofibroblast differentiation and other pathways. (Fig. 3B). The scRNA-Seq results showed that *grem1* was a characteristic gene expressed in the special subtype (Fig. 3C and D) and was typically expressed in the lesion area (Fig. 3E). Together, the single-cell sequencing and spatial transcriptome sequencing results obtained on the 7th and 56th days revealed that *grem1* may play an important role at the early pathological stage as the initiating factor (Additional file 1: Figure S2A and B). The authenticity and reliability of the results were verified by histochemical staining of mouse lung tissue sections. GREM1 was expressed at high levels at 7 days and colocalized with fibroblast markers (Fig. 3F). The changes in the *grem1* mRNA expression levels during the process of human idiopathic pulmonary fibrosis (IPF) were analyzed using the Gene Expression Omnibus (GEO) database, and the expression of *Grem1* in the group of patients with IPF was significantly higher than that in the healthy group (Fig. 3G), which indicated a role for *Grem1* in pulmonary fibrosis. Furthermore, we stimulated human pulmonary fibroblast-adult (HPF-a) cells with the optimal concentration of TGF-β1, 5 ng/ml (Additional file 1: Figure S3A-D), for verification in vitro and demonstrated that GREM1 expression first increased to a peak at 1 h and then decreased in a time-dependent manner (Fig. 3H and I). Recent studies have examined the function of GREM1 in fibrosis [19–23], but the mechanism of fibrosis caused by GREM1 remains unclear. Based on the signaling pathways related to the occurrence and development of inflammation and fibrosis (Fig. 3J), we analyzed the protein‒protein interaction (PPI) network of the genes in these signaling pathways to analyze the downstream targets of GREM1. GREM1 was correlated with bone morphogenetic protein (BMP) and PPP2R3A (Fig. 3K and S4A). GREM1 alters the pathological course of the disease through the BMP signaling pathway [19, 24, 25], but the mechanism through which GREM1 promotes the occurrence and development of disease by TGF-β1/PPP2R3A signaling is unclear. Studies have shown that PPP2R3A mainly regulates the cell cycle by targeting cell cycle regulators and apoptosis inhibitors [26], and this molecule has received extensive attention due to its involvement in the regulation of important tumor signaling pathways, developmental processes and the cell cycle. We concluded that PPP2R3A might be another downstream target of GREM1 that causes early pathological changes.

### PPP2R3A expression is induced in HPF-a cells after exposure to TGF-β1

**Fig. 4:**
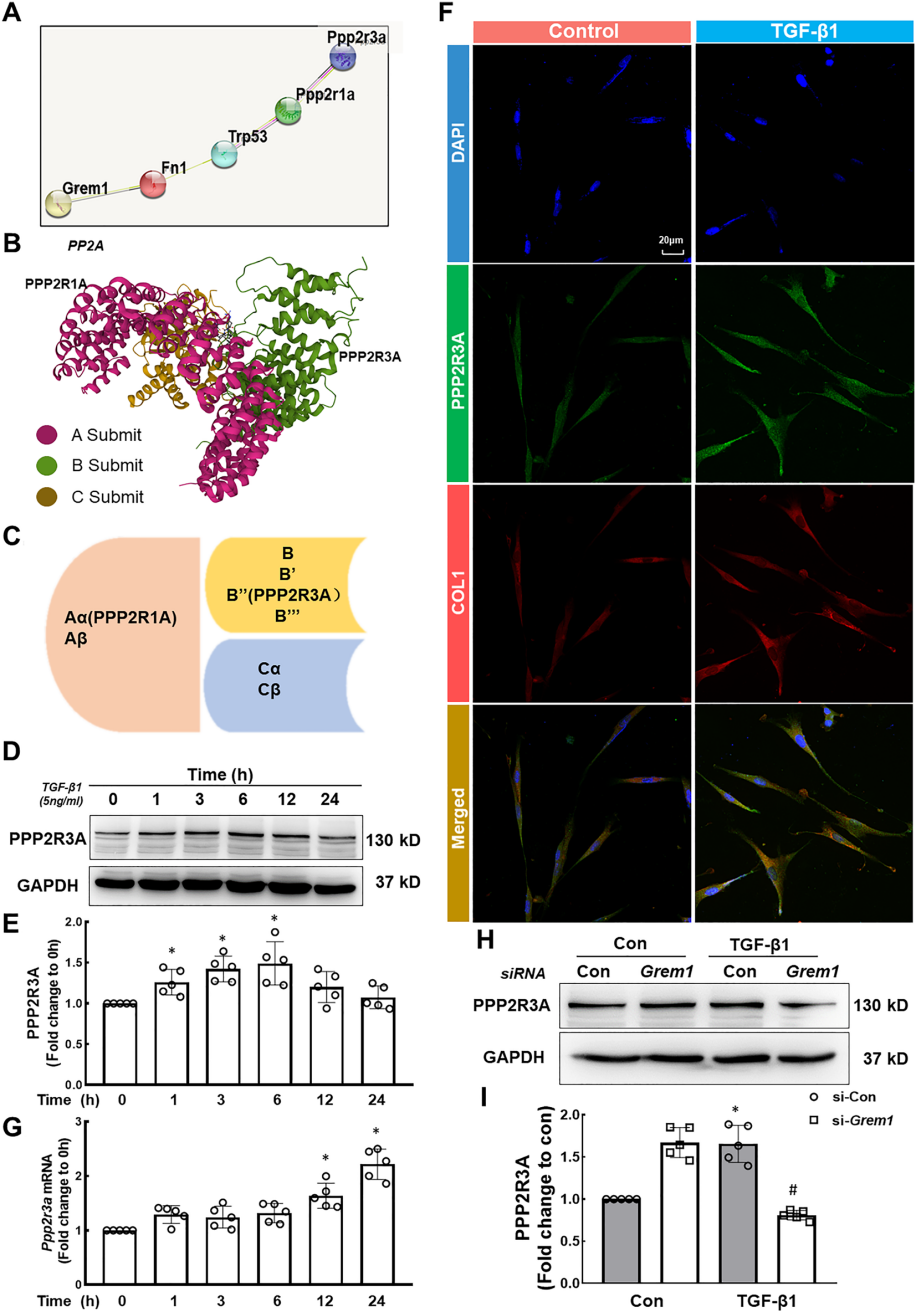
Effect of TGF-β1 on PPP2R3A expression in HPF-a cells and the relationship between GREM1 and PPP2R3A. **A** Protein interaction map of the main components investigated in our research. **B** Three-dimensional structure of the PP2A protein. **C** Location of each subunit of PP2A. **D**, **E** In HPF-a cells, the expression of PPP2R3A first increased and then decreased over time. The expression levels at 1, 3, and 6 h were significantly different from those at 0 h (*p < 0.05). **F** Representative images of immunofluorescence staining show that TGF-β1 treatment increased the expression of PPP2R3A protein in HPF-a cells; scale bar = 20 μm. **G** The *Ppp2r3a* mRNA level increased over time, and the expression levels at 12 and 24 h were significantly different from those at 0 h (*p < 0.05). **H**, **I** Representative Western blot results showing that *Grem1* knockdown partially reversed the upregulation of PPP2R3A induced by TGF-β1. *p < 0.05 indicates that the expression level of PPP2R3A in the si-Con group was higher than that in the control group after TGF-β1 treatment, showing successful establishment of the cell model. ^#^p < 0.05 indicates that PPP2R3A was expressed at a lower level in the si-*Grem1* group than in the si-Con group after TGF-β1 treatment

Follow-up studies involving an analysis of protein interactions were performed, as shown in Fig. 4A. Protein phosphatase 2A (PP2A) is a cellular serine/threonine protein phosphatase involved in various cellular processes and plays an important regulatory role in cell proliferation, differentiation and death [27]. Normally, the structural core subunit PP2Aa (PPP2R1A/PPP2R1B) interacts with the catalytic subunit PP2Ac (PPP2CA/PPP2CB) to form the core of the enzyme, and a broad range of regulatory B subunits (15 genes) interact with the core enzyme. The tissue specificity and substrate specificity of the PP2A holoenzyme complex were determined (Fig. 4B and C) [28]. PPP2R1A of the A subunit of PP2A promotes cell proliferation and migration and is a key fibrogenic factor [29], but the role of PPP2R3A of the B subunit in pathological processes remains unclear. Here, we mainly explored the role of PPP2R3A at the early stage of disease pathology and the mechanism through which GREM1 promotes pathological progression by TGF-β1/PPP2R3A signaling. HPF-a cells were treated with TGF-β1, and the results showed that PPP2R3A expression first increased, peaked at 6 h and then decreased over time (Fig. 4D and E). The immunofluorescence and qRT–PCR results were consistent with the Western blot (WB) results (Fig. 4F and G). The immunofluorescence results also showed that the morphology of the fibroblasts changed from the original spindle shape to an amoeba-like morphology after TGF-β1 stimulation (Fig. 4F). PPP2R3A expression was detected after the siRNA-mediated knockdown of *Grem1* (Additional file 1: Figure S4B and C), and *Grem1* knockdown partially reversed the increase in PPP2R3A expression induced by TGF-β1 (Fig. 4H and I), which suggested that PPP2R3A is a downstream target of GREM1. After validation in vivo, tissue immunofluorescence results suggested that PPP2R3A colocalizes with GREM1 in fibroblasts (Additional file 1: Figure S4D).

### PPP2R3A mediates the TGF-β1-induced proliferation and activation of HPF-a cells

**Fig. 5:**
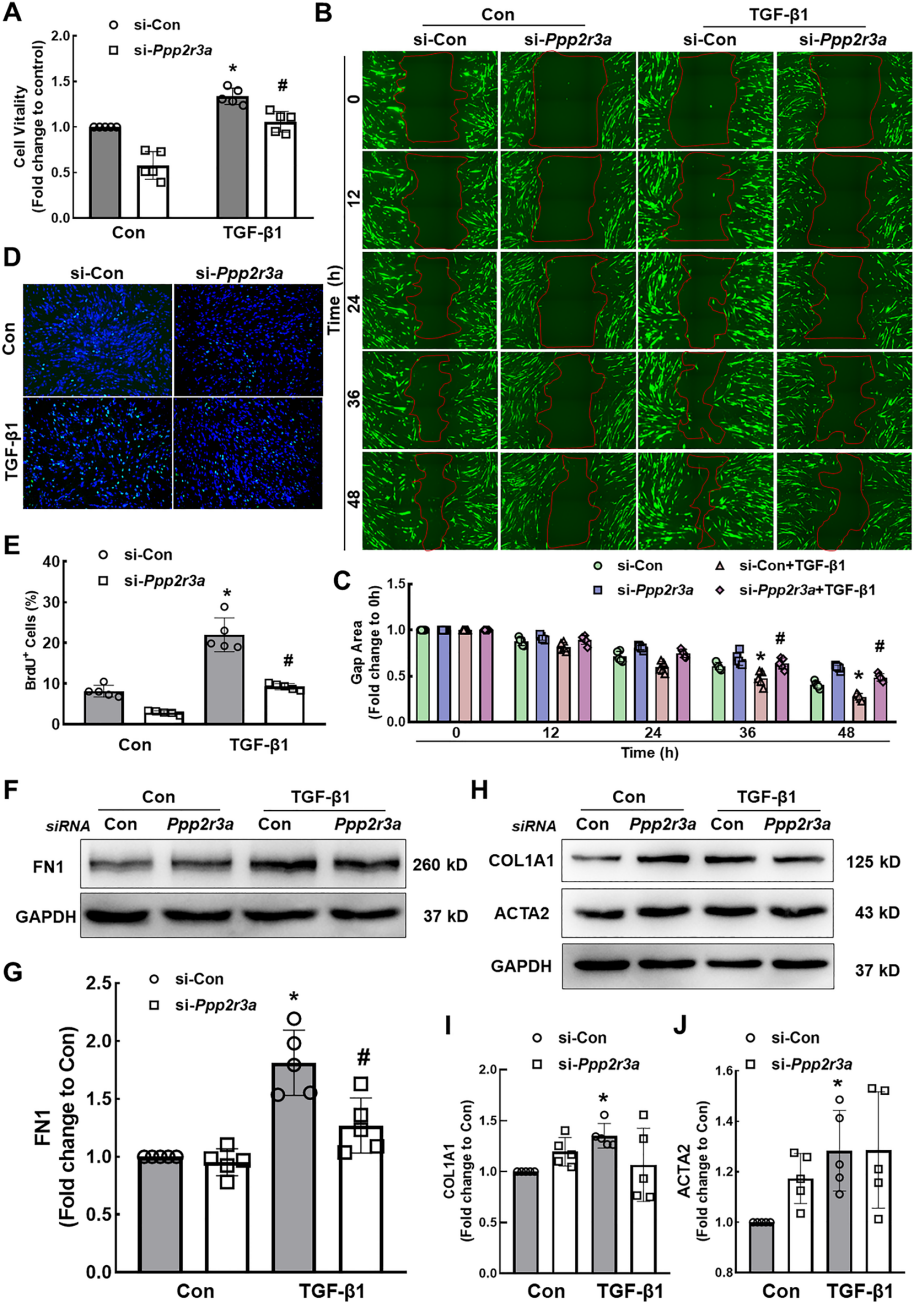
PPP2R3A mediates TGF-β1 signaling to induce the proliferation and activation of HPF-a cells. **A** CCK-8 assays show that *Ppp2r3a* knockdown partially reversed the increase in the viability of HPF-a cells induced by TGF-β1. *p < 0.05 indicates that the cell viability of the si-Con group after TGF-β1 treatment was higher than that in the control group and that the model was therefore successfully established. ^#^p < 0.05 indicates that the cell viability of the si-*Ppp2r3a* group was lower than that of the si-Con group after TGF-β1 treatment. **B**, **C** Wound healing experiments showed that the downregulation of *Ppp2r3a* expression attenuated cell migration induced by TGF-β1. *p < 0.05 indicates that the cell migration in the si-Con group after TGF-β1 treatment was higher than that in the control group and that the model was successfully established. ^#^p < 0.05 indicates that the cell migration of the si-*Ppp2r3a* group was lower than that of the si-Con group after TGF-β1 treatment. **D** The combined immunofluorescence images of BrdU (green) and DAPI (blue) show that the downregulation of *Ppp2r3a* expression attenuated cell proliferation induced by TGF-β1. **E** Percentage of BrdU-positive cells in five independent experiments. *p < 0.05 indicates that the cell proliferation of the si-Con group after TGF-β1 treatment was higher than that of the control group, which showed that the model was successfully established. ^#^p < 0.05 indicates that the cell proliferation of the si-*Ppp2r3a* group was lower than that of the si-Con group after TGF-β1 treatment. **F**, **G** Downregulation of *Ppp2r3a* expression partially reversed the increase in FN1 expression induced by TGF-β1. *p < 0.05 indicates that the expression of FN1 in the si-Con group after TGF-β1 treatment was higher than that in the control group and thus that the model was successfully established. ^#^p < 0.05 indicates that the expression of FN1 in the si-*Ppp2r3a* group was lower than that in the si-Con group after TGF-β1 treatment. **H** Downregulation of *Ppp2r3a* expression had little effect on the increase in Col1 and α-SMA expression induced by TGF-β1. **I**, **J** *p < 0.05 indicates that the expression of Col1 and α-SMA in the si-Con group after TGF-β1 treatment was higher than that in the control group, which revealed that the model was successfully established

Based on accumulating evidence, the migration, proliferation and activation of lung fibroblasts are the main causes of pulmonary fibrosis [30, 31], and thus, the effect of PPP2R3A on these cellular functions was investigated in HPF-a cells. TGF-β1 increased the expression of marker proteins related to fibrosis (Additional file 1: Figure S5A-D), induced fibroblast migration and increased fibroblast viability (Additional file 1: Figure S5E-G). However, the specific knockdown of *Ppp2r3a* (Additional file 1: Figure S6A-C) partially reversed the increases in cell viability, migration and proliferation (Fig. 5A−E) and specifically reversed the TGF-β1-induced increase in FN1 expression (Fig. 5F and G) but had little effect on the expression of COL1 and α-SMA (Fig. 5H−J).

### ***Upregulation of PPP2R3A in the lungs of mice exposed to SiO***_***2***_

**Fig. 6:**
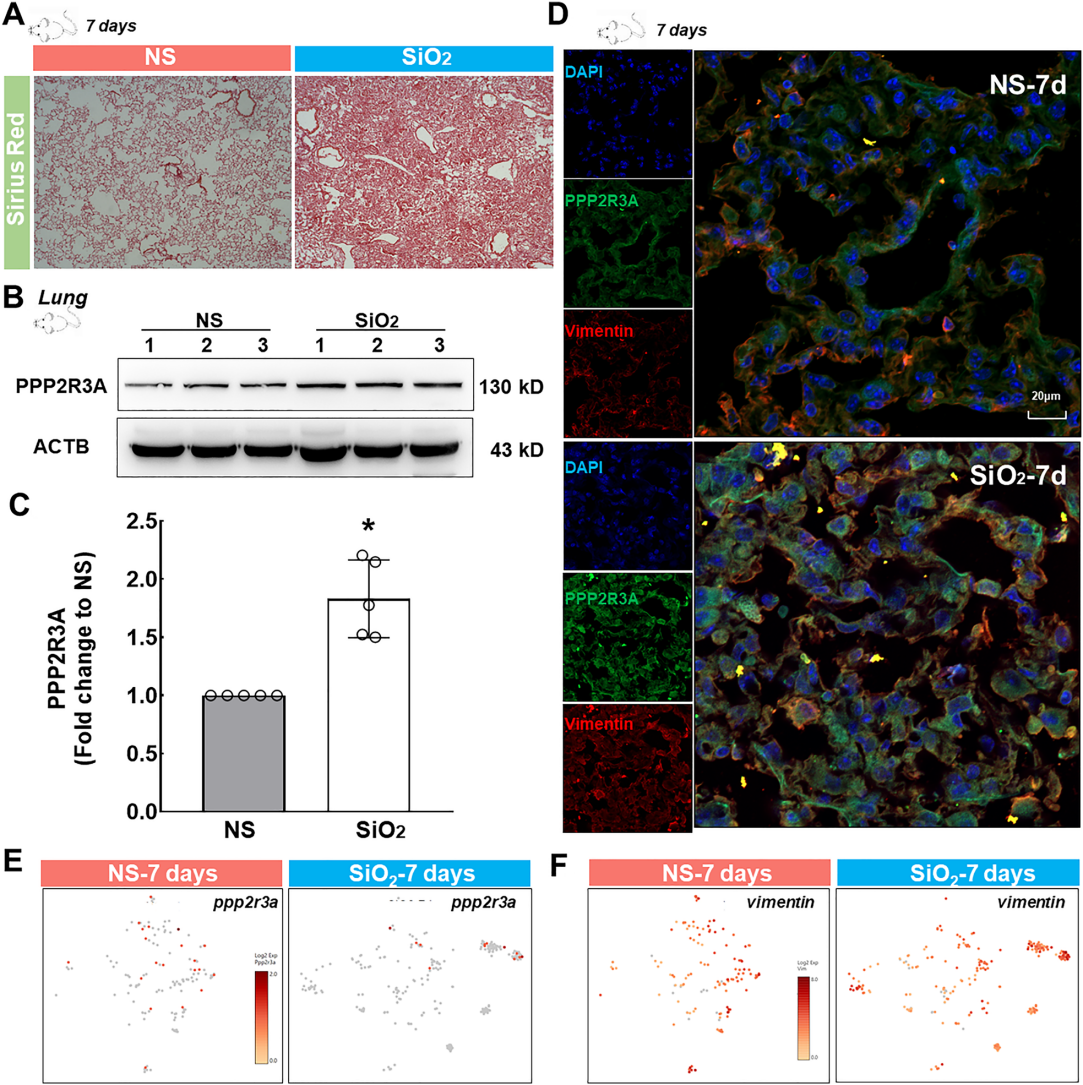
Mouse model of the early pathology of lung silicosis and expression of PPP2R3A in mouse lung tissue. **A** Sirius Red staining shows significantly greater collagen deposition in the lung tissue of the silica group than that of the saline group, which revealed that the model was successfully established. **B** Representative WB results showing higher PPP2R3A expression in the lung tissue of the silica group than that of the saline group. **C** *p < 0.05 indicates that the difference between the two groups is significant. **D** Immunohistochemical staining showing that PPP2R3A is expressed on fibroblasts. The expression level in the experimental group was higher than that in the control group. **E** The scRNA-Seq results show increased *ppp2r3a* expression in inflammatory-proliferative fibroblasts. **F** The scRNA-Seq results show that *vimentin* expression was increased in inflammatory-proliferative fibroblasts

Mice were exposed to SiO_2_ for 7 days to verify the in vitro observations based on scRNA-Seq results and the initiating role of PPP2R3A. Sirius red staining showed obvious collagen deposition with no typical formation of silicon nodules in the SiO_2_ group, indicating the successful establishment of a mouse silicosis model and initiation of fibrosis in the lung (Fig. 6A). The WB results showed higher expression of PPP2R3A in the SiO_2_ group than in the control group (Fig. 6B and C). Immunohistochemistry showed an increase in the level of Vimentin, a specific fibroblast marker, which indicated the proliferation of fibroblasts in the lung, and the PPP2R3A expression detected by immunohistochemistry was consistent with the WB results (Fig. 6D). Moreover, *ppp2r3a* was mainly expressed in resting fibroblasts in the control group (Fig. 6E). During the transformation from resting fibroblasts to inflammatory-proliferative fibroblasts after SiO_2_ treatment, *ppp2r3a* and *vimentin* were found to be expressed in inflammatory-proliferative fibroblasts in the SiO_2_ group (Fig. 6E and F).

## Discussion

Pulmonary fibrosis caused by silica inhalation is a major challenge for clinicians and a major problem in the field of public health due to the lack of specific targets for screening and diagnosis at the early stage and the lack of specific treatment measures at the later stage. In our study, we performed single-cell transcriptome sequencing to analyze and classify the subtypes of fibroblasts in the lung tissues of normal saline- and silica-treated mice. We identified a heterogeneous subtype of fibroblasts that was only detected in the silica group. Because the genes expressed in this subtype overlap with inflammatory fibroblasts, ECM fibroblasts and myofibroblasts, we defined this subtype as inflammatory-proliferative fibroblasts. According to recent studies, the occurrence of pulmonary fibrosis is due to the direct transdifferentiation of resting fibroblasts into inflammatory fibroblasts or ECM fibroblasts [18]. In our research, we found that cells at the resting state partially transdifferentiated into inflammatory-proliferative fibroblasts. Few studies have investigated the transdifferentiation of intermediate fibroblasts into inflammatory fibroblasts and ECM fibroblasts in pulmonary fibrosis. Blocking the conversion of resting fibroblasts into inflammatory-proliferative fibroblasts may slow or even reverse the early and progressive development of pathology.

We compared our scRNA-Seq data with recently published analyses of mouse and human lung fibroblast subtypes [6, 16], which mainly described myofibroblasts, resting fibroblasts, adipose fibroblasts, inflammatory fibroblasts and ECM fibroblasts. The resting fibroblast body is small and fusiform. Upon stimulation with inflammation and other factors, these cells transform into other types of fibroblasts and participate in repair after injury. Myofibroblasts express α-SMA and participate in the occurrence of fibrotic diseases [6]. Increased expression of α-SMA indicates fibroblast activation. The activation of resting fibroblasts is one of the main sources of myofibroblasts. Lipofibroblasts contain large cytoplasmic lipid droplet inclusions and unrestricted biofilms or lipid vacuoles and play important roles in lung development, surfactant synthesis and retinoic acid metabolism [32]. These cells generally do not change substantially during the onset of pulmonary fibrosis. Inflammatory fibroblasts and ECM fibroblasts are representative heterogeneous fibroblasts detected under pathological conditions and mainly induce inflammation, proliferation and migration. However, inflammatory-proliferative fibroblasts, which are cells at an intermediate state, were identified in this study and have not been previously reported. We found that inflammatory-proliferative fibroblasts play an important role in the process of transdifferentiation and promote cell proliferation and migration.

Newly emerged heterogeneous fibroblasts feature the expression of *grem1*, which reportedly promotes the migration and proliferation of normal lung cells [33, 34] and epithelial-mesenchymal transition (EMT) [35, 36] and regulate endothelial-mesenchymal transition (EndMT) [20], but the mechanism through which it initiates early pathological changes is unclear. A bioinformatics analysis showed that GREM1 was related to BMP and PP2A and that GREM1, PPP2R3A, BMP2, TGF-β1 and FN1 were enriched, as shown in Table 2 and Additional file 1: Figure S6D. BMP is a member of the TGF-β family that was first discovered due to its ability to induce bone formation. Defects in the BMP signaling pathway or its regulation are the basis of various human diseases. This pathway regulates cell proliferation, differentiation, migration, apoptosis and chemotaxis under different pathological conditions [37]. Previous studies have clearly shown that the BMP signaling pathway affects the occurrence and development of fibrosis [24, 38], mainly through the classic Smad-dependent pathway and the Smad-independent pathway [39]. Therefore, this study mainly explored the mechanism through which GREM1 promotes early pathological changes via PPP2R3A, a subunit of PP2A. PP2A is a major cellular serine-threonine phosphatase that has attracted attention due to its involvement in the regulation of important tumor signaling pathways, developmental processes, and the cell cycle [27, 28, 40]. Studies have demonstrated that specific knockout of the *ppp2r1a* gene (encoding the PP2A Aα subunit) in mice promotes inflammation and liver fibrosis [29]. Through a database analysis, we found that GREM1 is related to PPP2R3A in addition to PPP2R1A. PPP2R3A is a subunit of PP2A regulatory subunit B, also known as PR72/PR130. This molecule regulates the cell cycle mainly by targeting cell cycle regulators and apoptosis inhibitors and is the main regulator of cell proliferation. The scRNA-Seq results showed a slight increase in *ppp2r3a* expression, potentially due to increased expression in single cells rather than an increased number of cells expressing *ppp2r3a*. This conclusion is based on the number of dark red dots shown in Fig. 6E. The scRNA-Seq results showed that this molecule was expressed mainly in resting fibroblasts among normal mouse lung fibroblasts. In an early pathological model of tracheal silica instillation, *ppp2r3a* was expressed in inflammatory-proliferative fibroblasts. We postulate that although its overall level did not show major changes, this molecule still plays an important role in the fibroblast transdifferentiation process. Most researchers agree that a change in mRNA expression precedes a change in protein levels. However, our research showed that a change in the expression of the PPP2R3A protein preceded the change in mRNA expression. Many levels of regulation of gene expression may exist, and regulation at the transcriptional level is only one mechanism. Posttranscriptional, translational and posttranslational regulation may contribute to these findings. The results may be due to posttranslational regulation or positive regulation of transcriptional mechanisms. Because TGF-β and BMP play cross-regulatory, synergistic or antagonistic roles in multiple signaling pathways [41], we cannot exclude the possibility that the TGF-β1/GREM1/PPP2R3A pathway promotes pathological changes, whereas the TGF-β1/GREM1/BMP pathway can hinder the occurrence and development of fibrosis. However, the mechanism of action based on the two pathways is not discussed in this paper and will be studied in the future.

**Table 2:** The relationship between GREM1/BMP and TGF-β1/PPP2R3A

Pathway	Gene
Mesenchymal cell differentiation	Bmp2|Fn1|Tgfb1|Grem1
Regulation of the cellular response to growth factor stimulus	Bmp2|Tgfb1|Grem1
Extracellular matrix organization	Bmp2|Fn1|Tgfb1|Grem1
Regulation of the Wnt signaling pathway	Bmp2|Tgfb1|Grem1|Ppp2r3a
Wnt signaling pathway	Bmp2|Tgfb1|Grem1|Ppp2r3a
Cell–cell signaling by Wnt	Bmp2|Tgfb1|Grem1|Ppp2r3a
Positive regulation of the Wnt signaling pathway	Bmp2|Tgfb1|Ppp2r3a
Cell surface receptor signaling pathway involved in cell–cell signaling	Bmp2|Tgfb1|Grem1|Ppp2r3a
Regulation of the MAPK cascade	Bmp2|Fn1|Tgfb1|Grem1
Negative regulation of cell population proliferation	Bmp2|Tgfb1|Grem1
Regulation of cell adhesion	Bmp2|Fn1|Tgfb1|Grem1

Shows the related signaling pathways in which GREM1, BMP, TGF-β1, PPP2R3A and FN1 were enriched

FN1, COL1, and α-SMA are three markers of fibrosis. Previous studies have shown that FN1 mainly affects cell migration and proliferation [42]. COL1 is the main component of the ECM, and excess accumulation of ECM may impair lung function. COL1 mainly affects cell adhesion and migration [43]. α-SMA is the most commonly used molecular marker of smooth muscle cells and myofibroblasts. Myofibroblasts play an important role in fibrosis, and α-SMA is activated in these cells and is involved in both cell migration and proliferation [44–46]. However, we found that *Ppp2r3a* knockdown specifically affected the expression of the fibrotic marker FN1 but had little effect on COL1 and α-SMA expression, which suggested that PPP2R3A may specifically alter FN1 expression to cause early pathological changes. One previous study showed that the expression of the target protein specifically affects FN1 but has almost no effect on COL1 [32], similar to our findings. We performed a GO enrichment analysis of genes expressed in inflammatory-proliferative fibroblasts to further analyze the mechanisms and found that FN1 was differentially expressed at the early pathological stage and mainly enriched in inflammatory-proliferative pathways (Table 3), which may explain why PPP2R3A affected FN1 but not other fibrosis markers.

**Table 3:** Signaling pathways involving FN1 that contribute to the process of inflammation

Pathway	Gene
Positive regulation of cell adhesion	Fn1|Hsp90aa1|Ccn1|Cd74|Lgals1|Ccl2|Spp1|Thbs1
Inflammatory response	C3|Cebpb|Fn1|Hp|Ier3|Saa3|Ccl2|Ccl7|Serpina3n|Thbs1|Timp1
Response to wounding	C3|Fn1|Ccn1|Ccl2|Sod2|Serpine2|Thbs1|Timp1|Tnc
Regulation of fibroblast proliferation	Fn1|Fth1|Cd74|Sod2
Regulation of collagen biosynthesis	Fn1|Ccl2|Prdx5

Shows the signaling pathways that were enriched in the top 50 genes of the inflammatory-proliferative fibroblasts, including FN1 and inflammation-related signaling genes

## Conclusions

**Fig. 7:**
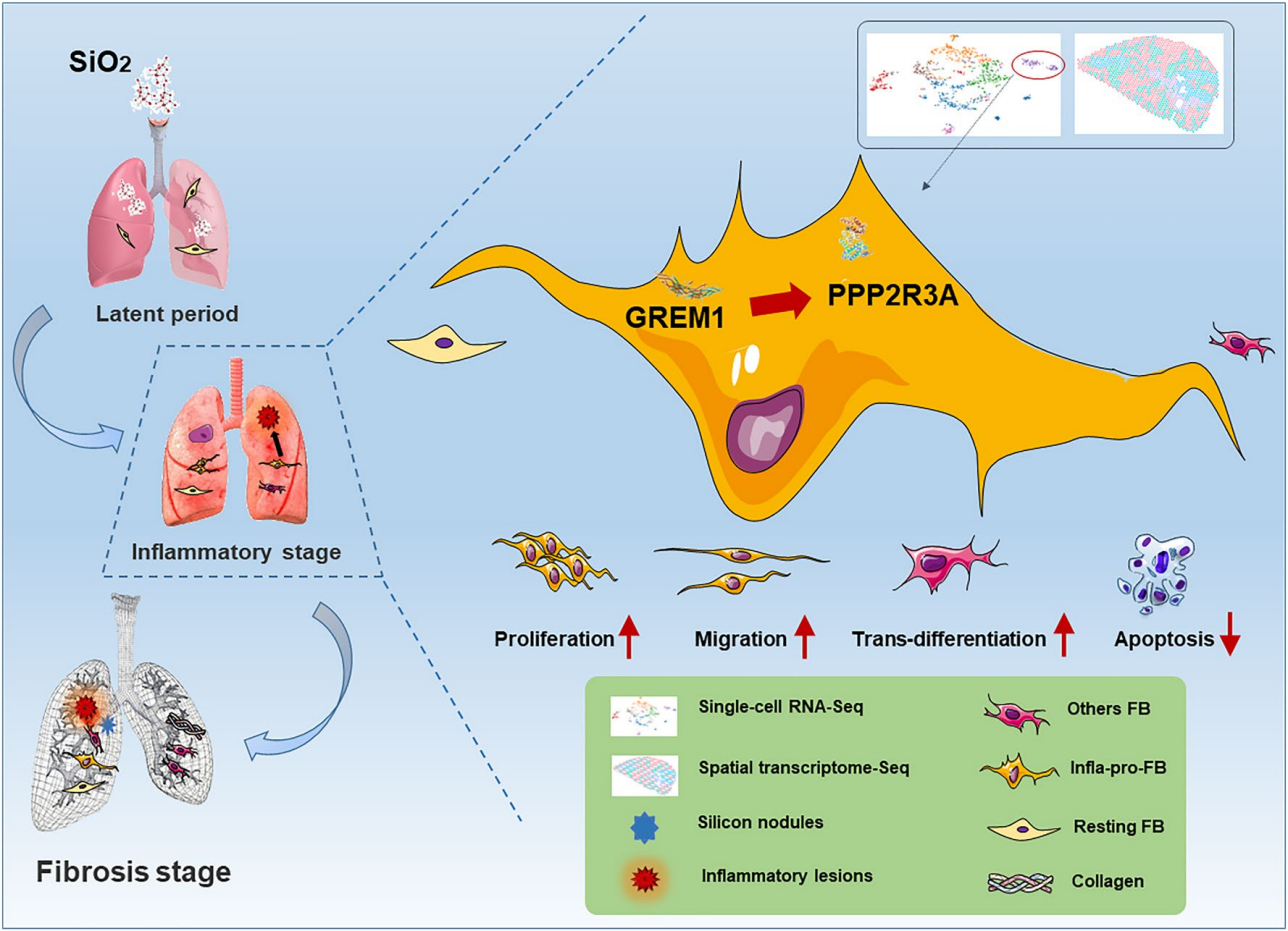
PPP2R3A affects the function and mechanism of heterogeneous fibroblasts (GREM1) during early pathological changes in the lung

In summary, in a model of the early pathology after tracheal silica infusion, inflammatory-proliferative fibroblasts indicated the occurrence and progression of the disease, and this type of heterogeneous fibroblasts was characterized by the expression of *grem1*, which may serve as a predictive biomarker of early pathology (Fig. 7). Moreover, we found that PPP2R3A was a downstream target of GREM1 and that its expression was related to early pathological regulation, which suggests that it may become a potential target in the early blockade of disease development.

## Materials and methods

### Reagents

SiO_2_ particles were purchased from Sigma–Aldrich Company (S5631; Billerica, MA, USA), and approximately 80% of the particles had a diameter of 1–5 μm. According to Stokes’ law, precipitation selection, acid hydrolysis, and baking were performed at 200 °C for at least 16 h. The silica sample was dissolved in normal saline and used to generate the mouse model of pulmonary fibrosis. Recombinant TGF-β1 protein was purchased from Nanjing GenScript Biotechnology Co., Ltd., and used to construct a cell fibrosis model. Primary antibodies against PPP2R3A (rabbit polyclonal antibody) were purchased from Proteintech, and GREM1 antibody (rabbit polyclonal antibody) was purchased from Shanghai Shenggong Biotechnology. Antibodies against GAPDH (mouse monoclonal antibody) were obtained from Bioworld, Inc. A calcium ion fluorescence probe (Fluo-4 AM) was purchased from Beyotime Biotechnology Company (China).

### Establishment of a mouse model of silicosis

Male C57BL/6 mice weighing 20–25 g were purchased from the Experimental Center of Nanjing Medical University. The mice were housed under a constant temperature (23 °C) and humidity (50%) with a 12-h light/12-h dark cycle and allowed to eat and drink freely. Animals of an appropriate age were anesthetized with an intraperitoneal injection of pentobarbital sodium, the trachea was surgically exposed, and a single dose of the prepared silica suspension (0.2 g/kg in 50 mg/ml saline) was injected into the trachea. The animals in the control group were administered the same volume of sterile normal saline. Lung tissues were collected 7 days after modeling. The lungs used for immunohistochemistry were first perfused with PBS, treated with 4% formalin, dehydrated with a 30% sucrose solution, sectioned and frozen for staining. All animal operations were performed in strict accordance with the ARRIVE guidelines, and the animal procedures were approved by the Institutional Animal Care and Use Committee of Southeast University School of Medicine.

### Single-cell RNA library construction and sequencing

We used Cell Ranger software (10 × Genomics) for the alignment of scRNA-Seq reads, collapsing of reads to unique molecular identifier (UMI) counts, cell calling, and depth normalization of the transcriptome libraries. We used the Chromium instrument and the Single Cell 3 Reagent kit (V1) to prepare individually barcoded scRNA-Seq libraries according to the manufacturer’s protocol (10X Genomics). The cells were then clustered according to the surface markers expressed on different cells and were divided into different subgroups. Gene comparison and naming were performed, names for each subgroup were defined, and Loupe Browser 5.0 was used to view and analyze the obtained data.

### Spatial transcriptome sequencing

Adult C57BL/6 mice were anesthetized, and lung tissues were removed. The samples were frozen and stored until sectioning. A tissue section was adhered to the surface of a glass slide, fixed and stained with standard hematoxylin and eosin. The lung tissue sample was then permeabilized and prepared into an information library containing barcodes. Subsequently, the cells were clustered, and the locations of different cells in different original spatial positions were determined.

### Cell culture

Human pulmonary fibroblast-adult (HPF-a) cells were purchased from ScienCell and cultured in DMEM supplemented with 10% fetal bovine serum, 100 U/ml penicillin, 100 μg/ml streptomycin and 2 mM L-GlutaMAX (Gibco). The cells were placed in a cell culture incubator with 5% CO_2_ and a temperature of 37 °C. For the experiments, we seeded the cells in a 24-well plate at a concentration of 1 × 10^5^ cells/ml and performed further processing after stabilization of the cell status. The cell concentration was adjusted for the different experiments according to the corresponding requirements.

### Western blotting

The protein levels in HPF-a cells and mouse lung tissues were detected by Western blotting, and the experimental results were imaged with a Tanon scanner. Briefly, HPF-a cells were cultured in a 24-well plate, treated with TGF-β1, and washed twice with PBS, and proteins were extracted with a cell lysis solution (100:1) containing protease inhibitors. Protein extraction from tissues was performed in a similar manner. After the tissues were ground, cell lysis buffer containing protease inhibitors was added, and the samples were lysed by overnight incubation at −80 °C. According to the reagent manufacturer’s protocol (Beyotime), the concentration of the extracted protein was determined using the BCA assay, the concentration was adjusted, loading buffer was added, and the sample was boiled at 100 °C for 5 min to denature the protein and successfully prepare the protein sample. The protein sample was separated by sodium dodecyl sulfate–polyacrylamide gel electrophoresis, transferred to PVDF membranes, and blocked with Tris-buffered saline containing 5% skim milk powder in Tween 20 (TBST) for 1 h at room temperature. The PVDF membrane was incubated with the primary antibody in a chromatography cabinet overnight (at least 16 h) at 4 °C. The next day, the PVDF membrane was washed 4 times with TBST and then incubated with the secondary antibody for 1 h at room temperature. The membrane was washed 3 times, covered with a luminescent solution and imaged with a Tanon scanner.

### Real-time quantitative PCR

The relative mRNA expression of *Grem1* and *Ppp2r3a* was detected by real-time quantitative PCR (qRT–PCR). HPF-a cells were plated according to the density needed for the experiment, and the corresponding treatments were administered after 24 h. After treatment, the cells were washed 3 times with RNase-free PBS, and total RNA was extracted from HPF-a cells using TRIzol reagent (Invitrogen) according to the manufacturer’s instructions. After total RNA extraction, the RNA concentration was measured with a NanoDrop One spectrophotometer (Thermo Fisher Scientific). Samples at different concentrations were normalized to contain approximately 400 ng of RNA and reverse transcribed into cDNAs. The cDNA samples were used as a template for qRT–PCR, and the cycle threshold (Ct) and ΔCt value were analyzed. Opticon monitoring software (Bio-Rad) was used for ΔΔCt quantification. The relative quantitation of mRNA expression was normalized to that of the endogenous reference (*Gapdh*).

### Immunofluorescence staining

Before the experiment, the cover glass was pretreated with polylysine, and the cells were then seeded in a 24-well plate containing the cover glass. After the experimental treatment, the medium in the 24-well plate was removed, and the cells were washed 3 times with PBS and fixed with 4% paraformaldehyde overnight at 4 °C. The next day, the paraformaldehyde was discarded, and the cells were washed 3 times with PBS, treated with 0.3% Triton X-100, blocked with goat serum at room temperature for 2 h, and incubated with the primary antibody overnight. On the third day, the cells were incubated with an appropriate fluorescent dye-conjugated secondary antibody (Alexa Fluor, Thermo Fisher Scientific) in the dark, the nucleus was stained with 4,6-diamino-2-phenylindole (DAPI), and images of the cells were captured using a fluorescence microscope. Immunofluorescence staining of mouse lung tissue sections was performed with the same steps as in the cellular immunofluorescence staining protocol after the paraformaldehyde was discarded.

### Wound healing experiment

A wound healing test was performed for the detection of cell migration. Specifically, HPF-a-GFP cells were seeded in a 24-well plate and cultured in a cell incubator until the cell density reached approximately 80%. A straight line of medium width was then drawn with the tip of a sterile 200-μl pipette tip. Similarly, a straight line was drawn perpendicular to the first line in each well to create a cross-shaped space. The medium was discarded, the wells were rinsed 3 times with sterile PBS to remove cell debris, and fresh standard medium was added to each well to ensure cell growth. The experimental group of cells was treated with 5 ng/ml TGF-β1, and we immediately collected digital images of the scratch gap (0 h) and then collected digital images at 12, 24, 36, and 48 h. We used ImageJ software to measure the area of the cell gap.

### CCK-8 assay

Cell viability was measured using the CCK-8 method (Dojindo, Tokyo, Japan) according to the manufacturer’s protocol. Briefly, after treatment of the cells, 10 μl of CCK-8 solution was added to each well of a 96-well plate, the plate was incubated at 37 °C for 1 h in the dark, and the absorbance was measured at 450 nm with a spectrophotometer. Cell viability was determined by calculating the survival of the experimental group relative to that of the control group. The percentage is shown.

### Bromodeoxyuridine labeling

The cells were plated on glass slides treated with polylysine, and after the cells grew to an appropriate density, TGF-β1 was added. Bromodeoxyuridine (BrdU) (Yeasen, 40204ES60) reagent was dissolved in PBS, and medium (1:1000) was added. After 4 h of incubation, the cells were fixed with 4% paraformaldehyde at 4 °C, washed 3 times with PBS, denatured with 2 N HCl/0.3% Triton X-100 at room temperature for 30 min, incubated with 0.1 M boric acid buffer (pH 8.0) for 10 min and blocked with goat serum at room temperature for 2 h. The cells were incubated with a BrdU antibody (1:100; SC-32323, Santa Cruz) at 4 °C overnight. After washing with PBS, the cells were incubated with appropriate fluorescent dye-conjugated secondary antibodies (Alexa Fluor, Thermo Fisher Scientific) in the dark for 2 h. The cells were then washed 3 times with PBS and mounted with mounting medium (Prolong Gold antifade reagent with DAPI; P36931, Life Technologies). The slides were imaged using a fluorescence microscope (Olympus IX70, Olympus America, Inc., Center Valley, PA, USA).

### Sirius red staining

After the mouse model was successfully generated, the lung tissues were fixed, removed and incubated with 4% paraformaldehyde. After sedimentation was completed, the lung tissues were sliced for use. The lung tissue sections were rinsed 3 times with PBS, incubated with Picrosirius red for 60 min at room temperature, quickly rinsed twice with an acetic acid solution, rinsed with absolute ethanol, soaked and dehydrated, mounted with neutral gum and stored at 4 °C. A microscope was used to capture bright-field images.

### RNA interference

Small interfering RNAs (siRNAs) were used to knock down the expression of proteins of interest. The siRNAs were purchased from Shanghai Jima Pharmaceutical Technology Co., Ltd., and the transfection reagent Lipofectamine 3000 was purchased from Thermo Fisher Scientific. We inoculated the cells in a 24-well plate and started transfection when the cell density reached 60–80%. At the beginning of the transfection experiment, we added the siRNA to one tube of serum-free medium and added transfection reagent to the other tube of serum-free medium. The samples were incubated for 5 min, and the two solutions were then mixed and incubated for 15 min. The solution was added to the wells and incubated for at least 12 h, and the standard medium was replaced. The cells were placed in an incubator at 37 °C for 24–72 h and used in subsequent experiments.

### Statistical analysis

The data are presented as the means ± standard deviations (SDs). Statistical analyses were performed with Student’s t test or one-way analysis of variance (ANOVA). P < 0.05 was defined as indicating significance.

## Supplementary Information

**Additional File 1: Fig. S1**. Classification of fibroblasts using single-cell transcriptome sequencing. **A** Markers of myofibroblast differentiation. **B** GO enrichment analysis of the molecular functions of the top 50 genes in Cluster 6. **C** GO enrichment analysis of biological processes related to the top 50 genes in Cluster 6. **Fig. S2**. Expression of *grem1* in saline- and silica-treated mice at 56 days determined by scRNA-seq and spatial transcriptome sequencing. **A** The expression of *grem1* in inflammatory-proliferative fibroblasts in the silica group at 56 days was higher than that in the normal saline group but lower than that at 7 days. **B** The spatial localization of *grem1* expression in the silica group was greater than that in the normal saline group but showed a decreasing trend compared with that at 7 days. **Fig. S3**. Optimal concentration of TGF-β1 for cell treatment. **A** A representative WB showed that a TGF-β1 concentration of 5 ng/ml yielded the highest expression of FN1, COL1, and α-SMA. **B** The statistical analysis of three experiments showed that the cells treated with 5 ng/ml TGF-β1 exhibited the highest expression of FN1. **C** The statistical analysis of three experiments showed that the cells treated with 5 ng/ml TGF-β1 exhibited the highest expression of COL1. **D** The statistical analysis of three experiments showed that the cells treated with 5 ng/ml TGF-β1 exhibited the highest expression of α-SMA. **Fig. S4**. Exploration of the downstream targets of GREM1 and verification of the *Grem1* knockdown efficiency. **A** KEGG analysis showing that GREM1 is related to BMP and PP2A in the TGF-β signaling pathway. Related research on PP2A is lacking. **B** The WB results show that among the three siRNAs, siRNA-*Grem1-485* exhibited the highest knockdown efficiency. **C** The qRT–PCR results showed that among the three siRNAs, siRNA-*Grem1-*485 was the most efficient, achieving approximately 70% knockdown. **D** The tissue immunofluorescence results suggest that PPP2R3A colocalizes with GREM1 in fibroblasts. **Fig. S5**. Cells were treated with TGF-β1 to construct a cell model, and TGF-β1 increased cell viability and migration. **A** A representative WB shows that the expression of the fibrosis-related markers FN1, COL1, and α-SMA increased in a time-dependent manner in the cells treated with the optimal TGF-β1 concentration of 5 ng/ml. **B** *p<0.05 indicates that the increase in FN1 expression from 0 h to the indicated time point was significant. **C** *p<0.05 indicates that the increase in COL1 expression from 0 h to the indicated time point was significant. **D** *p<0.05 indicates that the increase in α-SMA expression from 0 h to the indicated time point was significant. **E** The CCK-8 assay results show that TGF-β1 treatment increased the cell viability in a time-dependent manner to a peak at 72 h. *p<0.05 indicates that the difference in cell viability between 0 h and the indicated time point was significant. **F** The results of the wound healing experiment show that TGF-β1 treatment increased cell migration. **G** *p<0.05 indicates that the difference in cell migration between the TGF-β1 treatment group and the control group was significant. **Fig. S6**. Verification of the *Ppp2r3a *knockdown efficiency and GREM1 regulation of PPP2R3A through downstream signaling pathways. **A** The qRT–PCR results show that among the three siRNAs, siRNA-*Ppp2r3a-1174* exhibited the highest knockdown efficiency of approximately 70%. **B** The WB results show that among the three siRNAs, siRNA-*Ppp2r3a-1174 *exhibited the highest knockdown efficiency. **C** The statistical analysis of three experiments showed that siRNA-*Ppp2r3a-1174 *yielded the highest knockdown efficiency. **D** GO analysis of the signaling pathways enriched with GREM1, PPP2R3A, FN1 and p53. These results and those included in Table 2 indicate that GREM1 mainly regulates PPP2R3A through the Wnt signaling pathway.

## Data Availability

All of the relevant raw data and materials are freely available to any investigator upon request.
