# Supplementary material for: GREM1/PPP2R3A expression in heterogeneous fibroblasts initiates pulmonary fibrosis
Source: Cell Biosci. 2022 Aug 6;12:123. doi: 10.1186/s13578-022-00860-0 (PMC9356444; doi:10.1186/s13578-022-00860-0)
Supplement: Supplementary file 1 — Additional File 1: Fig. S1. Classification of fibroblasts using single-cell transcriptome sequencing. A Markers of myofibroblast differentiation. B GO enrichment analysis of the molecular functions of the top 50 genes in Cluster 6. C GO enrichment analysis of biological processes related to the top 50 genes in Cluster 6. Fig. S2. Expression of grem1 in saline- and silica-treated mice at 56 days determined by scRNA-seq and spatial transcriptome sequencing. A The expression of grem1 in inflammatory-proliferative fibroblasts in the silica group at 56 days was higher than that in the normal saline group but lower than that at 7 days. B The spatial localization of grem1 expression in the silica group was greater than that in the normal saline group but showed a decreasing trend compared with that at 7 days. Fig. S3. Optimal concentration of TGF-β1 for cell treatment. A A representative WB showed that a TGF-β1 concentration of 5 ng/ml yielded the highest expression of FN1, COL1, and α-SMA. B The statistical analysis of three experiments showed that the cells treated with 5 ng/ml TGF-β1 exhibited the highest expression of FN1. C The statistical analysis of three experiments showed that the cells treated with 5 ng/ml TGF-β1 exhibited the highest expression of COL1. D The statistical analysis of three experiments showed that the cells treated with 5 ng/ml TGF-β1 exhibited the highest expression of α-SMA. Fig. S4. Exploration of the downstream targets of GREM1 and verification of the Grem1 knockdown efficiency. A KEGG analysis showing that GREM1 is related to BMP and PP2A in the TGF-β signaling pathway. Related research on PP2A is lacking. B The WB results show that among the three siRNAs, siRNA-Grem1-485 exhibited the highest knockdown efficiency. C The qRT–PCR results showed that among the three siRNAs, siRNA-Grem1-485 was the most efficient, achieving approximately 70% knockdown. D The tissue immunofluorescence results suggest that PPP2R3A colocalizes with GREM1 in fibrobl [file 13578_2022_860_MOESM1_ESM.docx]

**Additional file Information for**

**GREM1/PPP2R3A expression in heterogeneous fibroblasts initiates pulmonary fibrosis**

Xiaoni Shi, Jing Wang, Xinxin Zhang, Shaoqi Yang, Wei Luo, Sha Wang, Jie Huang, Mengling Chen, Yusi Cheng, and Jie Chao

Supplementary Methods 2

Supplementary Figure S1 6

Supplementary Figure S2 8

Supplementary Figure S3 10

Supplementary Figure S4 11

Supplementary Figure S5 13

Supplementary Figure S6 15

Additional Methods

***Spatial transcriptomics (GSE183683)***

**1. Sample collection**

Obvious fibrotic lesions on CT scans of mice belonging to the model group indicated successful establishment of the model. Lung tissues were trimmed near the hilum in the horizontal direction and then immediately frozen in OCT on dry ice. These samples were stored at -80°C before proceeding to the next step.

**2. Spatial transcriptomic sequencing**

**2.1 Staining and imaging**

Cryosections were cut at a thickness of 10 μm with a cryostat (Leica, Germany) and mounted onto GEX arrays. The arrays were placed on a Thermocycler Adaptor with the active surface facing up, incubated for 1 min at 37°C, fixed for 30 min with methyl alcohol at -20°C, and stained with H&E. Bright-field images of the whole slide were acquired with a Leica DMI8 whole-slide scanner at 10x resolution.

**2.2 Permeabilization and reverse transcription**

Spatial gene expression analysis was performed using the Visium Spatial Gene Expression Slide and Reagent Kit (10x Genomics, PN-1000184). For each well, a slide cassette was used to create leakproof wells for adding reagents. Seventy microliters of permeabilization enzyme was added, and the samples were incubated at 37°C. For the NS-7 d, SiO_2_-7 d and NS-56 d samples, the incubation time was 24 min, but for the SiO_2_-56 d group, a 30-min incubation time was used due to severe lung fibrosis. Each well was washed with 100 μl of SSC, and 75 μl of RT master mix was added for cDNA synthesis (65°C for 15 min following by holding at 4°C).

**2.3 Preparation of the cDNA library for sequencing**

After first-strand synthesis, RT Master Mix was removed from the wells, 75 μl of 0.08 M KOH was added, and the plate was incubated for 5 min at room temperature. KOH was removed from the wells, and the cells were washed with 100 µl of EB buffer. Afterward, 75 μl of Second Strand Mix was added to each well for second-strand synthesis. The amplification of cDNAs (98°C for 3 min; 98°C for 15 s, 63°C for 20 s, 72°C for 1 min, 14 cycles; 72°C for 1 min; and hold at 4°C) was performed using a S1000^TM^ Touch Thermal Cycler (Bio-Rad). According to the manufacturer’s instructions, Visium spatial libraries were constructed using a Visium spatial library construction kit (10x Genomics, PN-1000184). The libraries were sequenced using an Illumina NovaSeq6000 sequencer with a sequencing depth of at least 100,000 reads per spot and a paired-end 150-bp (PE150) reading strategy (performed by CapitalBio Technology, Beijing).

**3. Analysis of spatial transcriptomics data**

A feature-barcode matrix was generated using 10X Space Ranger software, which processes, summarizes and aligns unique molecular identifier (UMI) counts against the mmu10 mouse reference genome for each spot. Only spots overlaying tissue sections were retained for further analysis. The unsupervised clustering analysis was performed using a graph-based algorithm with 10 principal components. The *t*-distributed stochastic neighborhood embedding (*t*-SNE) method was used to visualize the spots in two-dimensional space. Spatial feature expression plots were generated using Loupe Browser 4.1.0.

***Single-cell sequencing (GSE183682)***

**1. Sample collection**

In the model group (mice instilled with SiO_2_ suspension), mice with obvious high-density shadows on CT scans were included. Lung samples were collected from four mice for single-cell sequencing, and these samples were denoted NS-7 d, SiO_2_-7 d, NS-56 d, and SiO_2_-56 d samples. Whole lungs were removed from each mouse within 2 min of euthanasia and quickly washed three times with precooled PBS.

**2. scRNA-seq**

**2.1 Cell capture and cDNA synthesis**

Whole lung tissue was cut into small pieces (approximately 1 mm) and dissociated into single cells using a Lung Dissociation Kit (Miltenyi Biotech, 130-095-927, Germany). Using the Single-Cell 5' Library and Gel Bead Kit (10x Genomics, 1000169) and Chromium Single-Cell G Chip Kit (10x Genomics, 1000120), a cell suspension (300–600 living cells per microliter determined by CountStar) was loaded onto a Chromium single-cell controller (10x Genomics) to generate single-cell gel beads in emulsion (GEMs) according to the manufacturer’s protocol. Briefly, single cells were suspended in PBS containing 0.04% BSA. Approximately 20,000 cells were added to each channel, and the target cell recovery was estimated to equal approximately 10,000 cells. The captured cells were lysed, and the released RNA was barcoded through reverse transcription in individual GEMs. Reverse transcription was performed with a S1000^TM^ Touch Thermal Cycler (Bio-Rad) and the following temperature program: 53°C for 45 min, 85°C for 5 min and hold at 4°C. The cDNA templates were generated and then amplified, and the quality was assessed using an Agilent 4200 system (performed by CapitalBio Technology, Beijing).

**2.2 scRNA-seq library preparation**

The scRNA-seq libraries were constructed using the Single-Cell 5' Library and Gel Bead Kit, Single Cell V(D)J Enrichment Kit, Human T Cell (1000005) and Single Cell V(D)J Enrichment Kit according to the manufacturers’ instructions. The libraries were sequenced using an Illumina NovaSeq6000 sequencer with a sequencing depth of at least 100,000 reads per cell and a paired-end 150-bp (PE150) reading strategy (performed by CapitalBio Technology, Beijing).

**3. Data preprocessing**

**3.1 Analysis of scRNA-seq data**

Cell barcode filtering, alignment of reads and UMI counting were performed with Cell Ranger 4.0.0 software (https://www.10xgenomics.com/). The scRNA-seq data from four samples were combined using Cell Ranger aggr. Principal component analysis (PCA) was performed using the normalized data. Unsupervised clustering analysis was performed using Cell Ranger software with a graph-based algorithm. The top 10 principal components were used for clustering and *t*-SNE projections. The differential expression of genes between clusters was computed using sSEq- and edgeR-based methods. The GO and KEGG functional enrichment analyses of marker genes (FC≥2, P≤0.05) in Clusters 1 and 6 were performed using the R package Metascape.

**3.2 Cell type annotation**

Cell types were determined by clustering and marker gene expression.

Additional file Figure S1


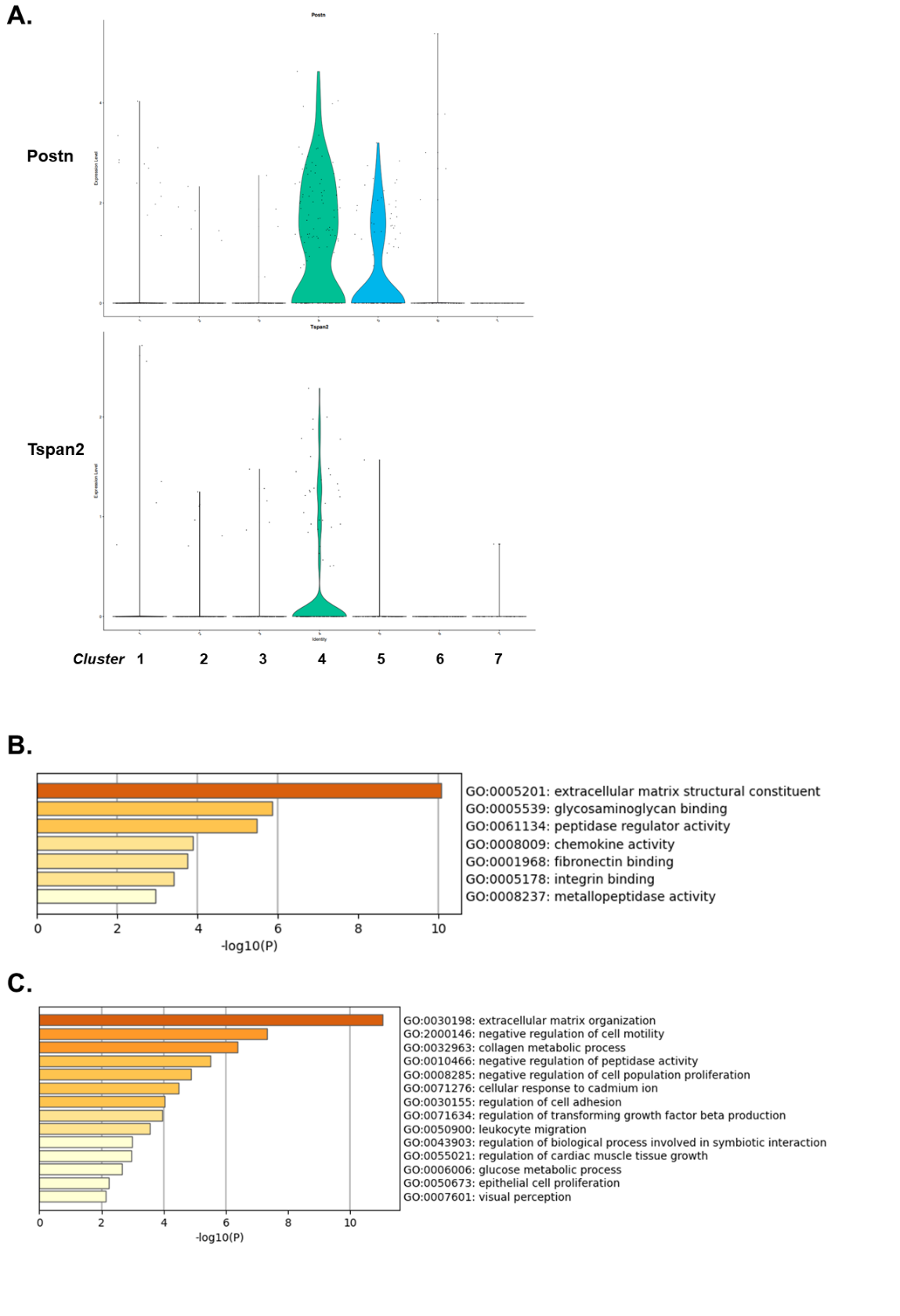


**Fig. S1. Classification of fibroblasts using single-cell transcriptome sequencing.** (A) Markers of myofibroblast differentiation. (B) GO enrichment analysis of the molecular functions of the top 50 genes in Cluster 6. (C) GO enrichment analysis of biological processes related to the top 50 genes in Cluster 6.

Additional file Figure S2


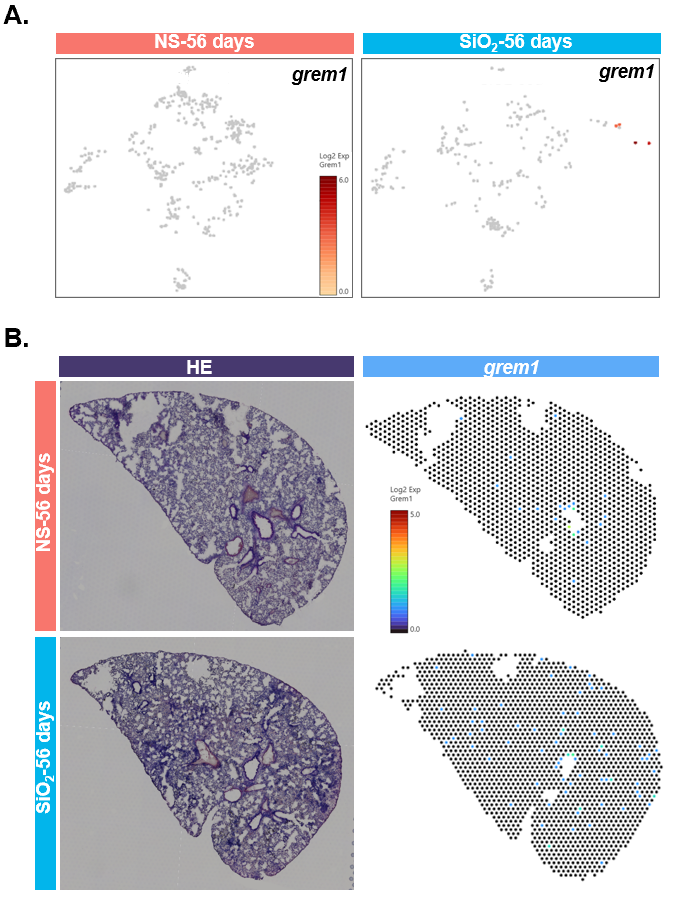


**Fig. S2. Expression of *grem1* in saline- and silica-treated mice at 56 days determined by scRNA-seq and spatial transcriptome sequencing.** (A) The expression of *grem1* in inflammatory-proliferative fibroblasts in the silica group at 56 days was higher than that in the normal saline group but lower than that at 7 days. (B) The spatial localization of *grem1* expression in the silica group was greater than that in the normal saline group but showed a decreasing trend compared with that at 7 days.

Additional file Figure S3


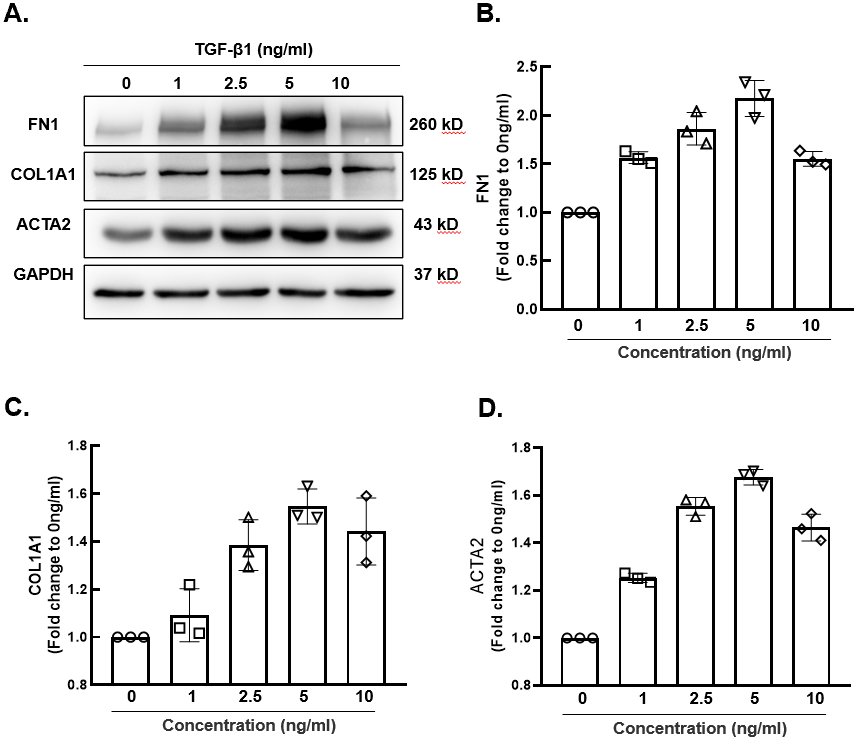


**Fig. S3. Optimal concentration of TGF-β1 for cell treatment.** (A) A representative WB showed that a TGF-β1 concentration of 5 ng/ml yielded the highest expression of FN1, COL1, and α-SMA. (B) The statistical analysis of three experiments showed that the cells treated with 5 ng/ml TGF-β1 exhibited the highest expression of FN1. (C) The statistical analysis of three experiments showed that the cells treated with 5 ng/ml TGF-β1 exhibited the highest expression of COL1. (D) The statistical analysis of three experiments showed that the cells treated with 5 ng/ml TGF-β1 exhibited the highest expression of α-SMA.

Additional file Figure S4


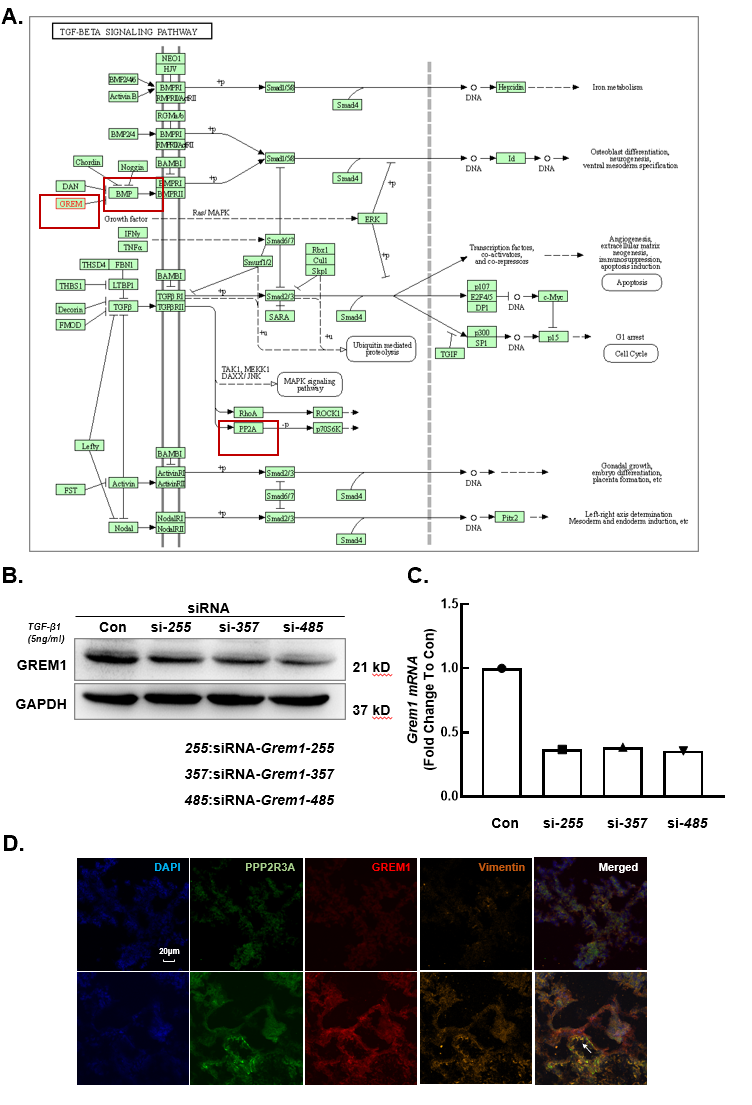


**Fig. S4.** **Exploration of the downstream targets of GREM1** **and verification of the *Grem1* knockdown efficiency.** (A) KEGG analysis showing that GREM1 is related to BMP and PP2A in the TGF-β signaling pathway. Related research on PP2A is lacking. (B) The WB results show that among the three siRNAs, siRNA-*Grem1-485* exhibited the highest knockdown efficiency. (C) The qRT–PCR results showed that among the three siRNAs, siRNA-*Grem1-*485 was the most efficient, achieving approximately 70% knockdown. (D) The tissue immunofluorescence results suggest that PPP2R3A colocalizes with GREM1 in fibroblasts.

Additional file Figure S5


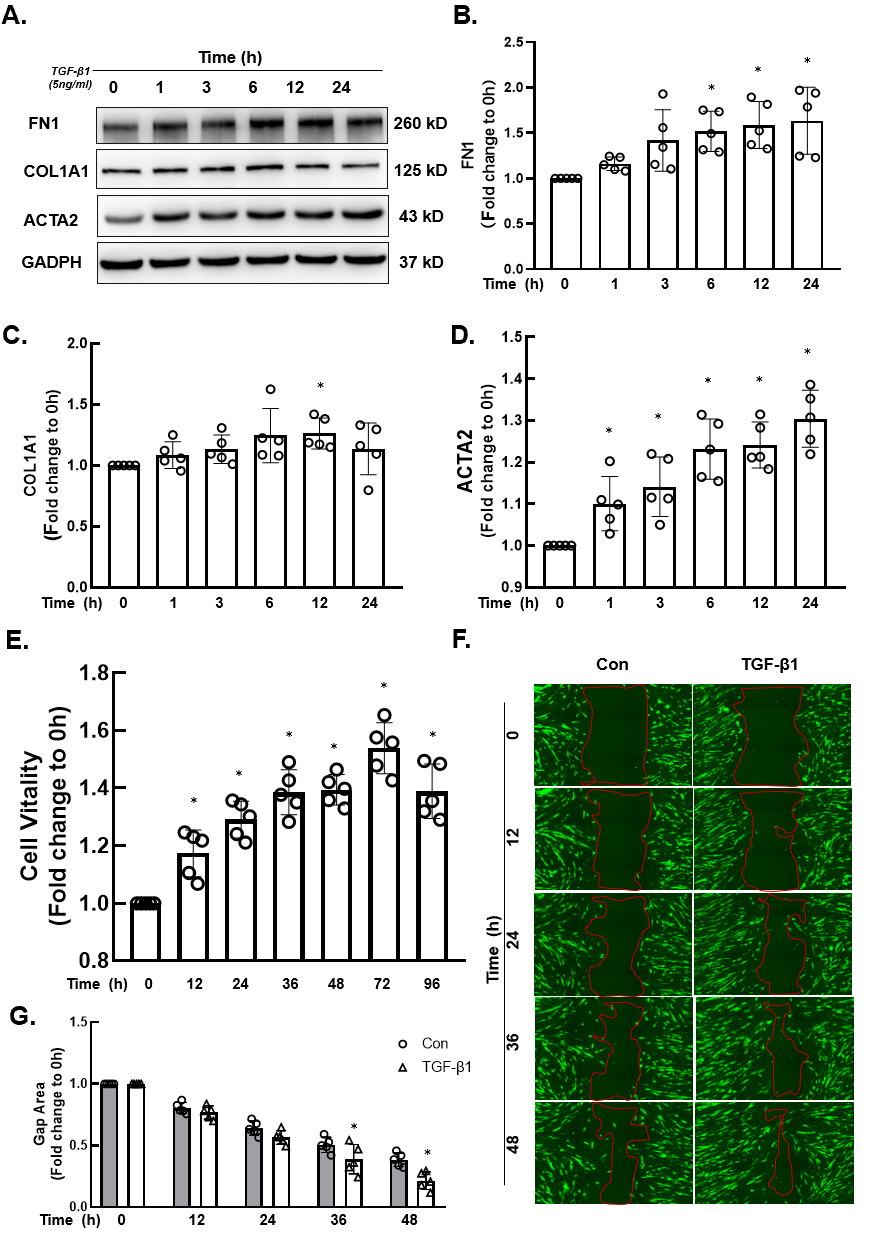


**Fig. S5. Cells were treated with TGF-β1 to construct a cell model,** **and** **TGF-β1 increased cell viability and migration.** (A) A representative WB shows that the expression of the fibrosis-related markers FN1, COL1, and α-SMA increased in a time-dependent manner in the cells treated with the optimal TGF-β1 concentration of 5 ng/ml. (B) *p<0.05 indicates that the increase in FN1 expression from 0 h to the indicated time point was significant. (C) *p<0.05 indicates that the increase in COL1 expression from 0 h to the indicated time point was significant. (D) *p<0.05 indicates that the increase in α-SMA expression from 0 h to the indicated time point was significant. (E) The CCK-8 assay results show that TGF-β1 treatment increased the cell viability in a time-dependent manner to a peak at 72 h. *p<0.05 indicates that the difference in cell viability between 0 h and the indicated time point was significant. (F) The results of the wound healing experiment show that TGF-β1 treatment increased cell migration. (G) *p<0.05 indicates that the difference in cell migration between the TGF-β1 treatment group and the control group was significant.

Additional file Figure S6


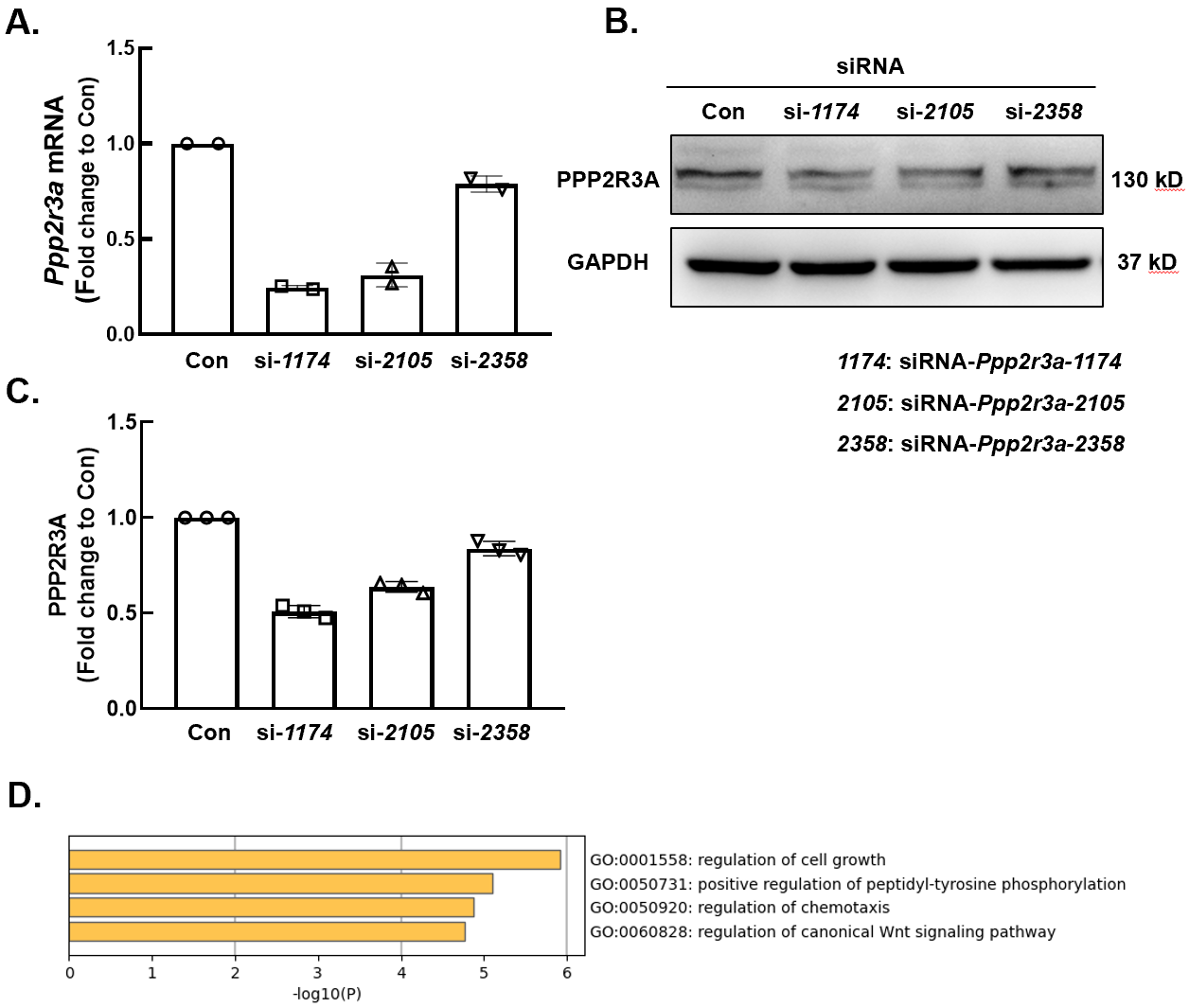


**Fig. S6. Verification of the *Ppp2r3a* knockdown efficiency and** **GREM1 regulation of PPP2R3A through downstream signaling pathways**. (A) The qRT–PCR results show that among the three siRNAs, siRNA-*Ppp2r3a-1174* exhibited the highest knockdown efficiency of approximately 70%. (B) The WB results show that among the three siRNAs, siRNA-*Ppp2r3a-1174* exhibited the highest knockdown efficiency. (C) The statistical analysis of three experiments showed that siRNA-*Ppp2r3a-1174* yielded the highest knockdown efficiency. (D) GO analysis of the signaling pathways enriched with GREM1, PPP2R3A, FN1 and p53. These results and those included in Table 2 indicate that GREM1 mainly regulates PPP2R3A through the Wnt signaling pathway
